# Editorial: Mechanisms and advances in respiratory allergic diseases

**DOI:** 10.3389/fimmu.2023.1186301

**Published:** 2023-03-28

**Authors:** Bao-Hui Cheng, Xiaojun Xiao, Yu Jin, Jing Li

**Affiliations:** ^1^ Department of Otolaryngology, Longgang E.N.T Hospital & Shenzhen Key Laboratory of E.N.T, Institute of Ear Nose Throat (E.N.T), Shenzhen, China; ^2^ Institute of Allergy and Immunology, Health Science Center, Shenzhen University, Shenzhen, China; ^3^ Department of Pathology, Yale University, New Haven, CT, United States; ^4^ Department of Allergy and Clinical Immunology, State Key Laboratory of Respiratory Disease, Guangzhou Institute of Respiratory Health, The First Affiliated Hospital of Guangzhou Medical University, Guangzhou, China

**Keywords:** allergic asthma, epithelial barrier dysfunction, inorganic particles, Aspergillus fumigatus, microbial dysbiosis

Allergic respiratory diseases (ARD), including asthma, chronic rhinosinusitis and allergic rhinitis, are amongst the most common allergies worldwide and constitute major public health and economic problem for their morbidity and impact on quality of life(1). A substantial documented study has been attributed to unraveling the mechanisms of respiratory allergic and inflammatory diseases, but still, a lot of questions are elusive. New findings in the pathogenesis of ARD which may provide new targets for diagnosis or treatment for ARD.

The lung epithelial barrier serves as a guardian towards environmental insults and responds to allergen encounter with a cascade of immune reactions that can possibly lead to inflammation(2). Epithelial barrier dysfunction is fascinating research area in the initial of allergic inflammation. Alessandrini et al. revealed that CYP1B1 deficiency leads to enhanced expression and activity of CYP1A1 in lung epithelial cells and to an increased availability of the AhR ligand kynurenic acid following allergen challenge. Thus, differential CYP1 family member expression and signaling *via* the AhR in epithelial cells represents an immunoregulatory layer protecting the lung from exacerbation of allergic airway inflammation. Wang et al. suggested a functional axis of AhR-TGF-β1 that is critical in driving allergic airway inflammation through regulating allergen-induced cellular autophagy. In addition, RNA-seq analysis suggests that autophagy is one of the major pathways and that CALCOCO2/NDP52 and S1009 are major autophagy-associated genes in AT2 cells that may contribute to the AhR-mediated cockroach allergen–induced airway inflammation and, subsequently, ARD.

Allergic respiratory diseases have increased dramatically due to air pollution over the past few decades. However, studies are limited on the effects of inorganic components and particulate matter with different particle sizes in smog on allergic diseases, and the possible molecular mechanism of inducing allergies has not been thoroughly studied. Yang et al. screened four common inorganic mineral elements (Al_2_O_3_, TiO_2_, Fe_2_O_3_, and SiO_2_) smog particles, and only 20 nm SiO_2_ particles significantly increased β-hexosaminidase release, based on dinitrophenol (DNP)-human serum albumin (HSA) stimulation, from IgE-sensitized mast cells. Yang et al. indicated that nano-SiO_2_ particles stimulation might synergistically activate IgE-sensitized mast cells by enhancing the MAPK signaling pathway and that nano-SiO_2_ particles exposure could exacerbate allergic inflammation.

As a common airborne allergen that contributes to allergic asthma, *Aspergillus fumigatus* (*A.f*) can colonize in the airway and lead to allergic bronchopulmonary aspergillosis (ABPA)(3). The pathogenesis of *A.f*-sensitized asthma and ABPA remains inadequate. Chen et al. suggested the distinct humoral and cell immunological responses in *A.f*-sensitized asthma and ABPA patients. ABPA patients have more severe eosinophilic inflammation and enhanced Th1 responses compared with *A.f*-sensitized asthma patients. Wang et al. supported the notion that miR-365-3p, which was diminished by IL-17 in murine and human asthmatic pathogenesis, functioned as an essential negative mediator in IL-17-stimuated inflammatory response by targeting ARRB2, which would shed new light to the understanding and therapeutics thereof of asthmatic inflammation.

Changes in microbiome (dysbiosis) contribute to severity of allergic asthma(4). Preexisting epidemiological studies in humans correlate perinatal dysbiosis with increased long-term asthma severity(5). However, these studies cannot discriminate between prenatal and postnatal effects of dysbiosis and suffer from a high variability of dysbiotic causes ranging from antibiotic treatment, delivery by caesarian section to early-life breastfeeding practices. Given that maternal antibiotic exposure in mice increases the risk of newborn bacterial pneumonia in offspring, Lingel et al. revealed that prenatally induced dysbiosis in mice led to an increase in pulmonary Th17^+^ non-conventional T cells with limited functional effect on airway resistance, pro-asthmatic Th2/Th17 cytokine production, pulmonary localization and cell-cell contacts. These data indicate that dysbiosis-related immune-modulation with long-term effects on asthma development occurs to a lesser extent prenatally and will allow to focus future studies on more decisive postnatal timeframes.

Pyroptosis and its mediated immune phenotype are crucial in the occurrence, development, and prognosis of asthma. Yang et al. demonstrated that BNIP3 was identified as a diagnostic marker and associated with immune cell infiltration such as, M2 macrophages. Small molecules obtained from the cMAP database that may have therapeutic effects on asthma are mainly DPP4 inhibitors. Yu et al. uncovered a previously uncharacterized role for the *de novo* creatine biosynthesis enzyme GATM in M2 macrophage polarization, which may be involved in the pathogenesis of related inflammatory diseases such as an T helper 2 (Th2)-associated allergic asthma. Kardas et al. summarized recent data on existing and potential monoclonal antibodies in asthma. Recent advances have resulted in the registration of a new antibody targeting TSLP (tezepelumab), with others being under development. In addition, as available monoclonal antibody treatments have shown little benefit among patients with T2-low asthma, research continues in this area, with several antibodies in development.

## Author contributions

BC drafted the initial draft. All authors carefully reviewed and approved the manuscript.
